# Understanding the pathogenesis of infectious diseases by single-cell RNA sequencing

**DOI:** 10.15698/mic2021.09.759

**Published:** 2021-08-04

**Authors:** Wanqiu Huang, Danni Wang, Yu-Feng Yao

**Affiliations:** 1 Laboratory of Bacterial Pathogenesis, Department of Microbiology and Immunology, Institutes of Medical Sciences, Shanghai Jiao Tong University School of Medicine, Shanghai 200025, China.; 2 Department of Infectious Diseases, Shanghai Ruijin Hospital, Shanghai 200025, China.

**Keywords:** single-cell RNA sequencing, infectious diseases, bacteria, viruses, fungi, parasites, immune response

## Abstract

Infections are highly orchestrated and dynamic processes, which involve both pathogen and host. Transcriptional profiling at the single-cell level enables the analysis of cell diversity, heterogeneity of the immune response, and detailed molecular mechanisms underlying infectious diseases caused by bacteria, viruses, fungi, and parasites. Herein, we highlight recent remarkable advances in single-cell RNA sequencing (scRNA-seq) technologies and their applications in the investigation of host-pathogen interactions, current challenges and potential prospects for disease treatment are discussed as well. We propose that with the aid of scRNA-seq, the mechanism of infectious diseases will be further revealed thus inspiring the development of novel interventions and therapies.

## INTRODUCTION

Infectious diseases have always been serious threats to public health. Excellent examples are the recent global epidemic of Coronavirus disease 2019 (COVID-19), long-standing influenza, HIV, *Salmonella*, as well as other bacterial infections. To clarify the pathogenesis of these diseases, it is necessary to understand the interactions between the host and the pathogen. Although traditional phenotypic measurements and bulk transcript analysis can provide some insights into pathogenesis, neither the heterogeneity of individual cell populations nor the pathogenic states can be appreciated. With the development of single-cell RNA sequencing (scRNA-seq), a new technique, which measures the transcriptome at the single-cell level, it is possible to study cell behaviors at a higher resolution. The use of scRNA-seq has refined the human cell landscape [1] and driven progress in various areas including immunology [2], developmental biology [3], oncology [4], and infectious diseases. Single cell approaches allow for identification of cellular heterogeneity during infections as well as subpopulation differences that influence outcomes and individual differences. Recent advances in scRNA-seq analysis of infections have provided for a new understanding of host-pathogen interactions. In this review, we briefly introduce technological improvements in scRNA-seq, highlight reports that interrogate microbial pathogenicity and host immune responsiveness at the single-cell level, describe the current limitations of scRNA-seq, and discuss exciting prospects for the study of infections and possible clinical application.

## THE TECHNOLOGY ADVANCES IN SINGLE-CELL RNA SEQUENCING

The first transcriptome analysis at the single-cell level was conducted by Tang *et al.* in 2009 [5]. Since then, this technology has been continuously improved to meet different needs, leading to the emergence of several novel methods such as: SMART-seq2 [6], Drop-seq [7], inDrop [8], CEL-seq2 [9, 10], and MARS-seq [11]. To date, scRNA-seq has developed into a mature workflow, including single cell isolation, cell lysis, conversion of RNA into cDNA with amplification, library construction, sequencing, and analysis of the high-throughput data. These new technologies were developed by improving key steps including cell separation, library construction, sequencing depth, and quality.

The emergence of the use of barcodes [12] and unique molecular identifiers (UMI) was a huge advance [13]. The single-cell tagged reverse transcription (STRT) sequencing, which first introduced cell-specific barcoding at the reverse transcription stage, enabled highly multiplexed analysis [12]. After that, the addition of UMIs identified each molecule in a population as distinct, as a random DNA sequence label or an aliquot of a complex mixture [14]. Multiple scRNA-seq methods such as CEL-seq, Drop-seq, and MARS-seq assess the combination of barcodes and UMIs, providing for high throughput and sensitivity. However, multiplexing cDNA amplification sacrifices full-length coverage. These methods profile only the 5'- or 3'-terminus of the transcripts. In contrast, SMART-seq2 does not use barcodes or UMIs. The cDNA libraries are generated from individual cells, providing full-length transcripts [6] that increase scalability and availability. A newly developed multiple annealing and dC-tailing-based quantitative single cell RNA sequencing (MATQ-seq) not only captures the full-length RNA and genuine biological variation between whole transcriptomes [15] but also adds UMIs reducing bias with higher sensitivity and lower technical noise.

Another improvement worth mentioning is the application of maturing sequencing platforms. Previous methods, e.g., CEL-seq [9], which was inefficient and error-prone, were mainly plate-based. CEL-seq2 [10] employs an automated microfluidic platform from Fluidigm (C1 platform). With MARS-seq, a high-throughput implementation of the original CEL-seq method [11], cells are sorted by fluorescence-activated cell sorting (FACS). The newly developed Drop-seq [7] and inDrop [8] use nanoliter droplets to capture single cells. For Microwell-seq, a high-throughput and low-cost platform, individual cells are trapped in an agarose microarray and mRNAs are captured with magnetic beads [16]. All these innovative platforms have improved cell sorting accuracy. The availability of commercial platforms such as the Chromium system from 10×Genomics improves scRNA-seq efficiency by automation and lowers cost as well.

Briefly, even though various technologies have been developed, it is necessary to carefully consider the most suitable method for analysis based on actual situations and experimental purposes. A comparative analysis of prominent scRNA-seq methods revealed that Drop-seq is more cost-efficient when quantifying the transcriptomes of large numbers of cells at low sequencing depth. Single cell RNA barcoding and sequencing (SCRB-seq), with massively parallel single-cell RNA sequencing (MARS-seq), is preferable when quantifying transcriptomes of fewer cells [17].

## BACTERIAL INFECTION

The outcomes of an infection are complicated interactions of the pathogen and the host involving multiple biological factors. Pathogen virulence and growth state, host immunity, diverse cell types, and tissue microenvironments all impact disease progression and antimicrobial treatment. ScRNA-seq has become a powerful tool to probe cell-to-cell variability and uncover both host and bacterial factors that influence the severity of infection. To date, many scRNA-Seq studies have been performed to investigate the host-pathogen interactions (**Table 1**).

**TABLE 1.** The applications of scRNA-seq in infection.

**Pathogen**	**Host/cell**	**Cell isolation**	**scRNA-seq method/platform**	**Key findings**	**Ref.**
*S*. Typhimurium	Mouse/Bone-marrow-derived macrophages (BMDMs)	FACS	SMART-seq	The induction of macrophage type I IFN response was correlated with the variable PhoPQ activity of invading bacteria.	[19]
	Mouse/BMDMs	FACS	Smart-seq2	Macrophages harboring non-growing *Salmonella* displayed proinflammatory M1 polarization state while macrophages containing growing bacteria turned into an M2-like anti-inflammatory expression program.	[20]
	Mouse/BMDMs	FACS	CEL-Seq2	Development of scDual-seq, that captured host and pathogen transcriptomes simultaneously.	[22]
	Human/Monocyte-derived dendritic cells (MoDCs)	FACS	SMART-seq2	Invasive *Salmonella* strain ST313 exploited discrete evasion strategies within infected and bystander MoDCs to mediate its dissemination *in vivo*.	[21]
	Human/PBMCs	FACS	10xGenomics	Development of a deconvolution algorithm for inferring cell-type specific infection responses from bulk measurements.	[98]
*M. tuberculosis*	Human/PBMCs	FACS	10xGenomics	Revealed a gradual depletion of a NK cell subset from HC LTBI and active TB.	[26]
	Human/Monocyte-derived macrophages (MDM)	Microwell	Seq-Well	Development of the Seq-well method and revealed distinct heterogeneity between macrophages exposed and unexposed to Mtb.	[24]
SARS-CoV-2	Human/*M. mulatta*/*M. fascicularis*/Mouse/multiple tissues, e.g*.*, lung, ileal, nasal	Microwell Droplets Microfluidic	Seq-Well Drop-Seq 10xGenomics	Identified ACE2 and TMPRSS2 co-expressing cells within lung type II pneumocytes, ileal absorptive enterocytes, and nasal goblet secretory cells. Discovered that ACE2 was a human ISG *in vitro*.	[38]
	Human/Bronchoalveolar lavage (BAL) samples	FACS Microfluidic	MARS-seq 10xGenomics	Development of the Viral-Track method to scan for viral RNA in scRNA-seq data and revealed the infection landscape of mild and severe COVID-19 patients.	[40]
	Human/A549 cells, Primary human bronchial epithelial cells	Microfluidic	ECCITE-seq 10xGenomics	Combined CRISPR screen with scRNA-seq, identified new host factors required for SARS-CoV-2 infection, increased cholesterol biosynthesis were related to reduced infection.	[49]
	Human/CD4^+^ T cells	Microfluidic	10xGenomics	Hospitalization was associated with increased cytotoxic Tfh and cytotoxic T helper cells and a reduction in regulatory T cells.	[45]
	Human/PBMCs	Microfluidic	10xGenomics	Aging induced the dysregulation of the immune system and increased gene expression associated with SARS-CoV-2 susceptibility.	[104]
	Human/B cells	Microfluidic	10xGenomics	Identified potent neutralizing antibodies from convalescent COVID-19 patients.	[50]
Influenza	Mouse/Lung	FACS	MARS-seq	Analyzed viral and host transcriptomes in the same single cell and revealed cellular heterogeneity and novel markers specific for influenza-infected cells.	[105]
	Human/A549 cells	Microfluidic	10xGenomics	Infections performed at high MOIs resulted in increased viral gene expression per cell and IFN lambda 1 (IFNL1) showed a widespread pattern of expression more reliant on paracrine signaling.	[106]
	Human/A549 cells	Microfluidic	10xGenomics	Demonstrated the intricate effects of defective viral genomes on host transcriptional responses.	[107]
	Mouse/Lung	Microfluidic	10xGenomics	Demonstrated that two waves of pro-inflammatory factors were released during IAV infection.	[54]
HIV	Human/CD4^+^ T cells	Microfluidic	Fluidigm C1	Cell state driven by T-cell receptor mediated cell activation was the main factor of transcriptional heterogeneity and was tested as a biomarker of HIV permissiveness.	[108]
	Human/CD4^+^ T cells	Microfluidic	10xGenomics	Expression of HIV proviruses within the latent reservoir were influenced by the host cell transcriptional program.	[109]
	Human/CD4^+^ T cells	Microfluidic	Fluidigm C1	Characterized cell heterogeneity during HIV latency and reactivation and identified transcriptional programs leading to successful reactivation of HIV expression.	[110]
	Human/PBMCs	Microwell	Seq-Well	Characterized multiple dynamic cellular responses and gene expression modules that varied by time and cell subsets during acute HIV infection.	[53]
Zika virus	Human/Neuroepithelial Stem Cells(NES cells)	Microfluidic	Fluidigm C1	Zika virus (ZIKV) infected NES cells and radial glia cells and induced mitochondrial sequestration of centrosomal phospho-TBK1, nucleoside analogs inhibited ZIKV replication.	[111]
	Human/Developing cortex	Microfluidic	Fluidigm C1	AXL was a candidate Zika entry receptor in neural stem cells and its expression was conserved in rodents and human cerebral organoid model systems.	[112]
	Mouse/Neuronal stem cells(NSCs)	Microfluidic	10xGenomics	Generated a fully immunocompetent mouse model of ZIKV infection and the NS4B G18R mutation in ZIKV likely acted through its ability to diminish IFN-β levels.	[113]
Dengue virus	Human/PBMCs	FACS	Smart-seq2	Identified cells with viral RNA from human patients and studied the molecular signatures preceding the development of severe dengue infection.	[114]
Ebola virus	Rhesus monkeys/PBMCs	Microwell	Seq-Well	Demonstrated that the EBOV tropism, replication dynamics, and elicited immune responses were mediated by viral infection related to cytokine signaling.	[115]
*P. chabaudi*	Mouse/CD4^+^ T cells	FACS Microfluidic	SmartSeq-2 Fluidigm C1	Reconstructed the developmental trajectories of Th1 and Tfh (T follicular helper) cells during blood-stage *Plasmodium* infection.	[70]
	Mouse/CD4^+^ T cells	FACS	Fluidigm C1	CD4^+^ T cell-derived MCSF regulated expansion and activation on of specific myeloid subsets.	[71]
*P. falciparum*	Human/B^+^ erythrocytes	Microfluidic	Fluidigm C1	Discovered undefined sex-specific genes as well as three distinct clusters of late-stage asexual parasites largely defined by stage-specific genes.	[116]
	Human/RBCs	Droplets	Drop-seq	Revealed the gene expression signature of sexual commitment that AP2-G^+^ mature schizonts specifically upregulated additional epigenetic regulators.	[60]
*P. falciparum* *P. berghei*	Mouse, Human/Red blood cells (RBCs)	FACS	Smart-seq2	Observed sharp transcriptional transitions at the asexual stage and discovered a set of sex-specific genes involved in sequestration of mature gametocytes.	[61]
*P. falciparum* *P. berghei* *P. knowlesi*	Human/RBCs	FACS Microfluidic	SMART-seq2 10xGenomics	Assembled a Malaria Cell Atlas that presented the transcriptomic profiles of individual *Plasmodium* parasites across all morphological life cycle stages.	[65]
*Trypanosoma brucei*	*Glossina morsitans morsitans*/salivary glands	Microfluidic	10xGenomics	Described proteins associated with the different parasite developmental stages in salivary glands and highlighted a family of nonvariant surface proteins associated with metacyclic parasites.	[117]
*Toxoplasma*	Human/Monocytes	Microfluidic	10xGenomics	Revealed that CD14^+^CD16^-^ monocytes were key regulators of human monocyte transcriptional response to *Toxoplasma*.	[118]
*C. albicans*	Human/PBMCs	Microfluidic	10xGenomics	Integrated GWAS with bulk and scRNA-seq, identified 27 *Candida*-response QTLs and revealed a role for *LY86* in the anti-*Candida* host response.	[77]

Abbreviations: *P. knowlesi*: *Plasmodium knowlesi*; FACS: Fluorescence-Activated Cell Sorting; GWAS: Genome-Wide Associated Studies; HC: healthy control; IAV: Influenza A virus; ISG: Interferon-Stimulated Gene; LTBI: latent TB infection; MCSF: Macrophage Stimulating Factor; *M. fascicularis*: *Macaca fascicularis*; *M. mulatta*: *Macaca mulatta*; MOI: Multiplicity Of Infection; Mtb: *Mycobacterium tuberculosis*; NK: natural killer; PBMCs: Peripheral Blood Mononuclear Cells; *P. berghei*: *Plasmodium berghei*; *P. chabaudi*: *Plasmodium chabaudi*; *P. falciparum: Plasmodium falciparum*; *P. knowlesi*: *Plasmodium knowlesi*; QTL: Quantitative Trait Loci; SARS-CoV-2: Severe Acute Respiratory Syndrome Corona Virus 2; TB: tuberculosis.

One well-studied example of infectious diseases is *Salmonella* infection, a common food-borne pathogen that can produce acute or chronic symptoms, either a limited gastroenteritis or a systemic infection [18]. Recent studies have shown that the intracellular bacterial heterogeneity is influenced by host cell microenvironments which, in turn, can produce differential host cell immune responses. During the infection, macrophages are favorable niches for *Salmonella* survival and proliferation. The scRNA-seq expression profiling of infected macrophages revealed that the induction of a macrophage type I interferon (IFN) response correlated with variable PhoPQ activity of invading *Salmonella* [19]. When macrophages came across a subset of bacteria with highly modified lipopolysaccharides by PhoPQ, the macrophages tended to have a high type I IFN response. Meanwhile, macrophages harboring non-growing *Salmonella* displayed hallmarks of the proinflammatory M1 polarization state that differed little from bystander cells [20]. The non-growing bacteria did not trigger additional immune recognition by intracellular receptors. However, intracellular growing bacteria induced an M2-like anti-inflammatory response in macrophages, indicating that intracellular *Salmonella* were capable of escaping from the host defense by reprogramming macrophage polarization [20]. These data suggest that gene expression heterogeneity among infected cells creates diverse environments for *Salmonella* to either persist or exploit its host. In addition, the bacterial pathogenicity also plays an important role in regulating the host cell state and immune response.

Furthermore, different *S*. Typhimurium strains have been shown to produce marked differences during infection [21]. Compared with non-invasive *Salmonella*, the highly invasive and multi-drug resistant *S*. Typhimurium strain ST313 could produce a heterogeneous innate immune response that exploited divergent evasion strategies for dissemination *in vivo*. MoDCs infected with invasive *Salmonella* differentially regulated genes associated with endosomal trafficking and antigen presentation pathways. Invasive *Salmonella* induced higher expression of *IL10* and *MARCH1* but lower expression of CD83, allowing evasion of adaptive immune supervision.

Moreover, investigations have restricted analysis to only eukaryotic transcripts, thereby losing the ability to decipher the heterogeneity of both host and bacteria at the same time. The development of scDual-Seq enables the capture of host and pathogen transcriptomes simultaneously at the single cell level [22]. By utilizing this method to study the process of individual macrophages, the authors showed the rate of *S.* Typhimurium infection was non-uniform, supportive by the evidence showing the co-existence of all three cell subpopulations. These three cell states also showed evidence for a linear progression through consecutive stages of infection. However, the experiments were limited in that a high multiplicity of infection (MOI) could cause a variable number of bacteria in infected macrophages, thus masking certain infectious stages and phenotypes.

Another suitable model for studying host-bacterial interaction is *Mycobacterium tuberculosis* (Mtb), which sickens millions of people with tuberculosis (TB). Lungs infected with Mtb contain several coexisting lesion types such as solid cellular granulomas of densely packed macrophages and necrotic granulomas with an outer ring of T and B lymphocytes [23]. Technological advances in scRNA-seq helped to reveal the heterogeneity between different cell types and subpopulations during Mtb infection [24]. Gene expression shifts associated with cell growth and metabolism were found among cell clusters in response to Mtb. The complex dynamic of host and bacteria can result in distinct symptoms from latent TB infection (LTBI), in which patients remain clinically asymptomatic, to active TB, a contagious state in which patients may suffer from cough, fever and night sweats [25]. A small proportion of LBTI patients undergo progression into active TB. For better TB control, it is necessary to identify these populations by monitoring reliable biomarkers. Since the intensity of immune response and alterations in immune cell composition are critical indicators of severity of disease, many efforts have been made to investigated the transcript profiles of immune cells in peripheral blood and lesions. A recent scRNA-seq study compared the transcriptomes of peripheral blood mononuclear cells (PBMC) from healthy controls (HC), (LTBI and active TB patients. The results showed that there was a gradual depletion of the cytotoxic natural killer (NK) cell subset (CD3^−^CD7^+^GZMB^+^) during these three states [26]. The subset frequency also increased after anti-TB treatment, which confirmed that the frequency change in NK cells was involved in host disease severity and could be used as a novel biomarker to discriminate patients with TB from LTBI and HC.

The immune response to infectious agents is an orchestrated process that involves numerous cell types and pathways. Ronnie *et al.* comprehensively characterized the initial 48 h of the innate immune response to diverse pathogens by MARS-seq [27]. They found that most lymph node cell types showed little pathogen specificity and that antigen-specific immunity was driven by antigen-carrying dendritic cells and monocytes. The NK-driven IFN-γ response initiated a monocyte-specific signaling cascade that promoted Th1 development. Taken together, these data demonstrate an innate immune heterogeneity in response to a wide range of pathogens. This knowledge may provide insight into the development of safe and effective vaccines.

## VIRAL INFECTION

Viral infections are always of urgent public concern because of their high degree of transmissibility and pathogenic severity. Representative examples are influenza, HIV, and SARS-CoV. Advances in single cell technology coupled with mathematical modeling and computer science provide new horizons for understanding virus and host interactions.

COVID-19, a newly emerged severe acute respiratory syndrome coronavirus 2 (SARS-CoV-2), has become an ongoing global health emergency. Upon entry into the host cell, SARS-CoV-2 can replicate quickly and trigger a strong immune response leading to an acute respiratory syndrome, pulmonary tissue damage, and multiple organ failure [28]. This highly contagious disease progresses rapidly and so far has caused tens of millions of cases. Therefore, it is an urgent need to determine the pathogenic basis of this disease in order to improve existing prevention and treatment strategies. Single cell technology has shown its unique advantages in relevant investigations that uncovered sensitive cell types and the heterogeneity of the host immune response, each of which provided valuable insights into vaccine development.

**Figure 1:**
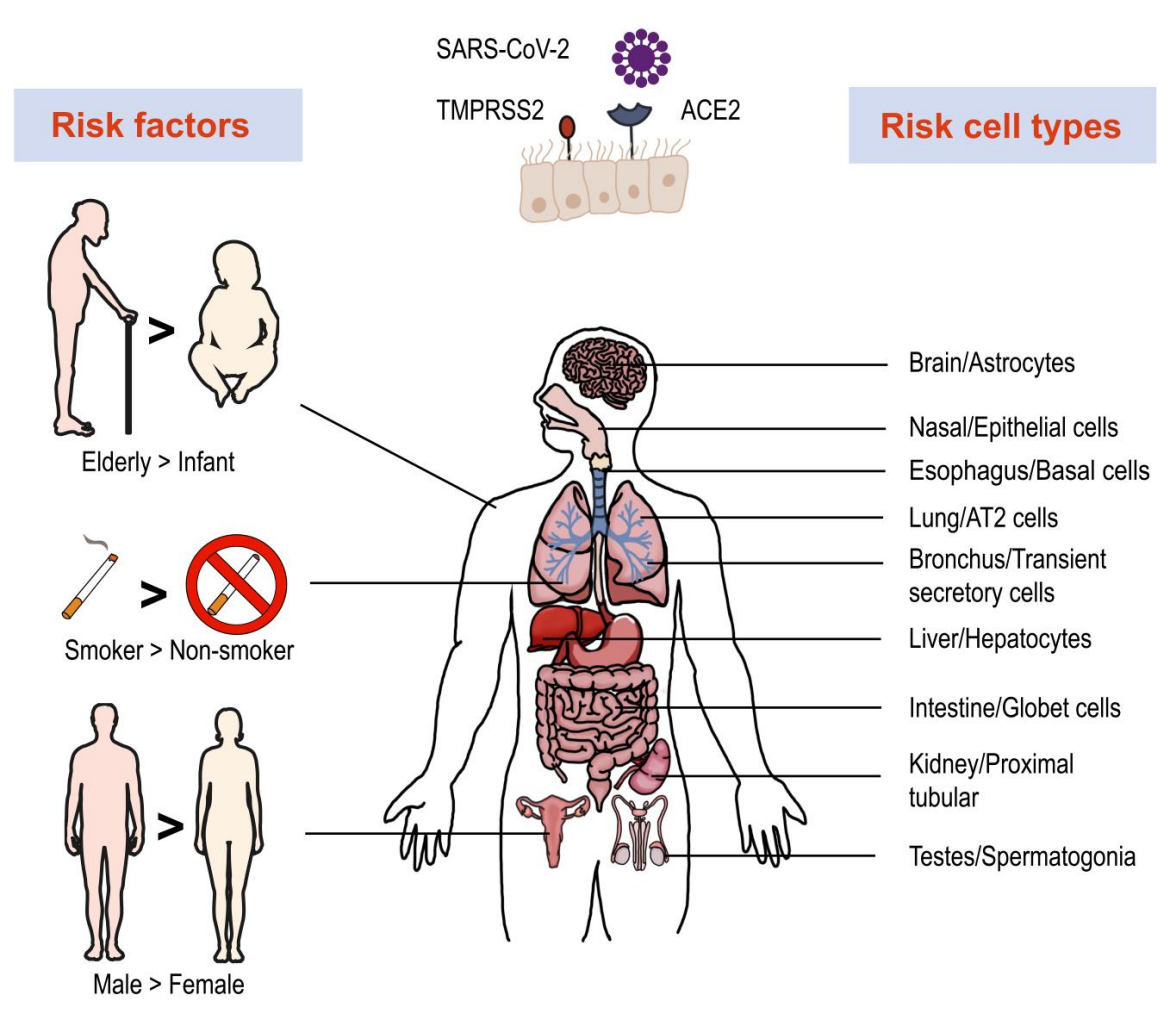
FIGURE 1: Risk factors, vulnerable organs and cell types involved in SARS-Cov-2 infection. Single-cell RNA-seq revealed high expression of ACE2 and TMPRSS2 in multiple organs and cell types, which are more vulnerable to SARS-Cov-2 infection. Several factors play important roles in infection and disease process, such as age, smoking and gender.

To invade cells, SARS-CoV-2 binds host angiotensin-converting enzyme 2 (ACE2) with its spike (S) protein and utilizes a type II transmembrane serine protease 2 (TMPRSS2) for priming and activation of the protein [29, 30]. Using scRNA-seq expression analysis of ACE2 and TMPRSS2, different cell types, tissues, and organs were assessed. The lung, kidney, bladder, and ileum were found to have the greatest expression [31]. Within these organs, oral mucosa [32], nasal epithelial cells [33], type II alveolar cells [31], and bronchial transient secretory cells [34] expressed the highest levels of ACE2 and TMPRSS2. As such, these cells have increased sensitivity to SARS-CoV-2 infection. Based on evidence obtained from a single cell RNA expression map of human coronavirus entry factors, spermatogonia and prostate endocrine cells were permissive to SARS-CoV-2 infection [34], suggesting that males were more vulnerable to coronavirus infection [35]. Multiple risk factors like age, gender and cigarette smoking contribute to the infection progression. The chronic smoke exposure increases lung ACE2 expression and triggers the expansion of ACE2^+^ secretory cells in the respiratory tract [35]. Aging increases the gene expression associated with SARS-CoV-2 susceptibility and COVID-19 can promote age-induced immune cell polarization [36]. The expression level of *TMPRSS2* is increased in older adults compare to children, which could be a reason for the higher risk of severe disease among the aged [37]. Furthermore, several studies demonstrated ACE2 to be an IFN-stimulated gene in humans that is upregulated by viral infection [38]. These data allow for the construction of a risk map of vulnerable cell types, revealing underlying virus transmission mechanisms (**Figure 1**).

COVID-19 patients present a wide spectrum of clinical manifestations, which range from asymptomatic infection to severe pneumonia [39]. The relationship between disease severity and the host immune response is not fully understood. Single cell analysis of immune cells and local landscape signature provide for an understanding of the dynamic immune response, which has great heterogeneity at the cellular level and differences among individuals.

A newly developed computational framework, Viral-Track, enables the global scanning of unmapped viral RNA in scRNA-seq data. Integrating these data with the host transcriptome distinguishes infected cells from bystander cells and manifests specific virus-induced expression [40]. This method was applied to bronchoalveolar-lavage (BAL) samples from severe and mild COVID-19 patients and revealed the dramatic diversity of immune cell compartments and responses. For example, the myeloid compartment in mild patients was enriched in alveolar macrophage and plasmacytoid dendritic cells (pDCs). In severe patients, accumulated myeloid cells were neutrophils, FCN^+^ monocytes, and monocyte-derived macrophages, which expressed a viral hallmark IFN type I response [41] and upregulation of inflammatory chemokine genes, e.g., CCL18. Similarly, severe COVID-19 infections were shown to be associated with developing neutrophils [42, 43] and abundant proinflammatory monocyte-derived macrophages [44] with an imbalance of the T cell compartment and T cell function. Proliferating cytotoxic CD8^+^ T cells and CD4^+^ follicular helper T cells (Tfh) were common in COVID-19 patients with reduced proportions of regulatory T cells (Treg) [45]. In moderate cases of COVID-19, CD8^+^ T cells were markedly expanded with higher levels of effector molecules, e.g., XCL1 [44], and a naïve phenotype for CD4^+^ T cells in severe cases [40].

In addition to the virus-stimulated alterations in cell composition, specific host immune response pathways provide insight into pathogenesis. SARS-CoV-2 infection induced the activation of the STAT1/IRF3 pathway and increased the expression levels of *IL6R* (interleukin 6 receptor) and *IL6ST* (interleukin 6 signal transducer), which may have a synergistic effect with elevated IL-6 to induce a strong inflammatory response [46]. These results provide evidence that cytokine inhibition may be a reliable strategy to attenuate severe inflammatory responses in COVID-19 patients, e.g., disruption of IL-6 and IL-6R binding [47], CCR1 and/or CCR5 pathway [48]. Additionally, host factors and pathways involved in key elements of the SARS-CoV-2 viral life cycle have been shown to have a role in regulating the infection [49]. For example, the ATPase proton pump which interacts with SARS-CoV-2 non-structural protein 6 (nsp6) and RAB7A which interacts strongly with non-structural protein 7 (nsp7). The loss of RAB7A reduced viral entry by sequestering ACE2 receptors via altered endosomal trafficking. The upregulation of cholesterol biosynthesis pathway with the small molecule amlodipine led to reduction of viral infection. It is possible that changes in lipid composition directly impacted SARS-CoV-2 virion maturation and infectivity, indicating potential new therapeutic targets [49].

Now that many patients have recovered from the virus infection, studying the immune system of these people will provide valuable information for prevention and treatments. Neutralizing antibodies are a powerful weapon to block virus entry. Through analyzing single B cell RNA/VDJ data of COVID-19 convalescent patients, novel potent neutralizing antibodies and S protein-binding antibodies have been identified, among which BD-368-2 showed a great therapeutic effect and GD1-69 showed a powerful neutralizing activity [50, 51]. Multiple investigations have demonstrated antibody cocktails to largely prevent viral mutation escape which produces an optimal antiviral effect [52]. ScRNA-seq technology enables the discovery of new neutralizing antibodies, accelerating development of new antiviral drugs and vaccines.

Individual cellular immunity participating in virus-host interaction is crucial for controlling virus elimination. A recent single cell transcriptome analysis revealed a dynamic cellular program during longitude HIV acute infection, such as NK cell expansion, naïve CD4^+^ T cell differentiation, and a rapid rise in plasma viremia coupled with cell-type specific interferon-stimulated gene (ISG) upregulation [53]. These cell type frequency shifts at different time points may shed light on how the immune response were orchestrated according to the infection progression. In accordance with changes in cellular phenotype, the release of pro-inflammatory factors also showed a time-course fluctuation [54]. During the early stage (day 1-3 post infection) of influenza A virus (IAV) infection, a group of PD-L^+^ neutrophils was the major contributor to the first wave of pro-inflammatory factors including Ccl3, Cxcl10, TNF-α, and IL1α. High level of virus HA mRNA and protein were also detected in the cell population. The second wave was mainly generated by another subset of Pf4^+^-macrophages at a later stage (day 7 post infection), which may be the precursor of alveolar macrophages. Enhancing the immune response against pathogens and reducing the cytokine storm are the major therapeutic aims of infectious disease. Analysis of these dynamic changes at single cell level could give deep insights into a more complete understanding of immunopathogenesis and offer possible targets for clinical treatment.

## PARASITIC INFECTION

Parasitic infection is an important worldwide cause of human disease such as Chagas disease, African trypanosomiasis, amebiasis, leishmaniasis, ascariasis, and schistosomiasis, leading to millions of deaths [55]. Malaria is a prevalent parasitic disease with 229 million victims worldwide in 2019 as reported by the World Health Organization (WHO). The disease is caused by a unicellular eukaryotic parasite, *Plasmodium* spp., that is transmitted to humans by *Anopheles* spp. mosquitoes [56]. *Plasmodium falciparum* is the most virulent cause of malaria. During human infection, the parasite attacks the liver and then invades the vasculature where red blood cells are infected resulting in an intra-erythrocytic development stage [57]. During this blood stage, the fast asexual-replication of parasites produces the clinical manifestation of the disease. The parasite then converts to a non-replicating sexual gametocyte form, which can be transmitted to female mosquito during a blood meal [58]. Understanding the complex life cycle of *Plasmodium* is fundamental for pathogen elimination and disease treatment. Although these distinct parasitic stages have been investigated at the overall population level [57], little is known about the variation between individual parasites.

ScRNA-seq promises a precise examination of transcriptional expression heterogeneity during parasitic stage switching and immune response. The transcriptional activation of AP2-G is known as the master regulator to initialize the sexual commitment [59]. AP2-G^+^ mature schizonts specifically upregulated epigenetic regulators like histone-modifying enzymes, which facilitated the subsequent gametocyte development [60]. Individual parasite transcripts showed distinct stage-specific transcriptional transitions and revealed differential routes *P. falciparum* converted into gametocytes [61–63]. A set of sex-specific genes involved in sequestrating gametocytes were found to function during immune evasion as well as transmission from human to mosquitos. Meanwhile, coupled with the synchronized single-cell transcriptome of infected red blood cells, novel gene signature that could be used as an indication to discriminate between sexual and asexual stages were identified [64]. In brief, scRNA-seq has allowed for high-resolution mapping of the life cycle of *Plasmodium* spp., providing a fundamental resource for the investigation of parasite biology and the study of malaria pathogenesis [65].

During the blood-stage of *Plasmodium* infection, CD4^+^ T cells and myeloid cells both play important roles in controlling the progress of disease, in which the intercellular communication fosters the immune regulation [66]. The CD4^+^ T-effector cell TCRβ repertoires undergo a polyclonal expansion dominated by TRBV3 gene usage in the acute phase of *P. chabaudi* infection [67]. Two types of CD4^+^ T cells are the main sources of protecting against malaria: (1) T helper (Th1) cells that secret IFN-γ and stimulate phagocytic cells to capture and kill parasites [68], and (2) Tfh cells that promote development of antigen-specific B cells that produce anti-parasitic antibodies [69]. During the activation and differentiation of CD4^+^ T cells, myeloid cells were shown to play a vital regulatory role, not only the dendritic cells presented the antigen stimulation, but inflammatory monocytes could also support a Th1 fate at the Th1/Tfh bifurcation [70]. Furthermore, CD4^+^ T cell-derived macrophage stimulating factor (MCSF) facilitated the expansion and activation of specific myeloid subsets such as macrophages during *P. chabaudi* infection, which contributed to parasite clearance and infection control [71]. Heterogeneity revealed by scRNA-seq provides insights into the key molecular and immunological mechanisms of parasite infection as well as the identification of potential targets for parasite elimination and vaccine development [72].

## FUNGAL INFECTION

Fungal disease is also a threatening heath problem, causing over 1.5 million deaths worldwide each year [73]. With advances in medical and surgical therapy of many different diseases, opportunistic fungi have become a major source of nosocomial infections especially among immunocompromised patients [74]. Candidiasis and aspergillosis account for a large proportion of these infections [75]. Candidiasis is caused by *Candida*, an opportunistic fungus that colonizes the skin and mucosa, which can lead to bloodstream infections, known as candidemia [76]. Single cell transcriptomic analysis showed that *Candida* stimulation induced dramatic and differential gene expression within CD4^+^ T cells, NK cells, and monocytes, while the upregulation of the IFN I pathway was consistent across all cell types [77]. Combination with published bulk RNA-seq data revealed that *Candida*-response expression quantitative trait loci (eQTL) was associated with disease susceptibility. Of note, *LY86* was found to be a potential candidemia-risk allele, with reduction of *LY86* weakening the migration of monocytes, thus increasing susceptibility for candidemia [77].

Nevertheless, the use of scRNA-seq to determine the transcriptomes of pathogens and hosts during fungal infection are still in their infancy. Most investigations employ single cell transcriptomics that focus on life cycles, cell states, taxonomic diversities, and ecological interactions [78, 79], rather than fungal infection. Given the increasing morbidity and mortality of fungal disease, deeper and more detailed explorations of fungal-host interactions are urgently needed.

## CHALLENGES AND LIMITATIONS

Although single cell technology has revolutionized many fields, there are still several challenges impeding deep exploration. A major limiting factor is the amount of available nucleic acid in that one mammalian cell contains only 10 pg of total RNA [80]. This limit may result in artificial zeros either systematically or accidently, thus hindering complete downstream analysis [81]. Using computational statistical models, it may be possible to circumvent this limitation by modulation of sample variation and noise by use of VIPER [82] and scSDAEs [83]. With regard to infection, most studies have focused on either the host or the pathogen, whereas dual RNA-seq allows analysis of the transcripts of both host and bacteria simultaneously [84], but not at the single-cell bacterial level. However, improved scDual RNA-seq does enable profiling of both host and bacterial transcripts in one individually infected mammalian cell [22]. It is important to note that the detection of intracellular bacteria is technically difficult since a single bacterium contains only 100 fg of total RNA. Further, the mRNA of bacteria and some virus like hepatitis C virus and dengue virus lack poly (A) tails which are required for library construction. Enzymatic addition of poly (A) tails, adaptor ligation, and/or random priming for amplification [22, 85] address this problem but also require additional steps to deplete redundant rRNA [86]. Advanced methodologies for characterization of single infected cells of both host and pathogen are needed.

Infection is a complicated process, with outcomes of infection dependent on the heterogeneity of host and pathogen. Host heterogeneity is at the cell type level, in other words, the vulnerability of host cells and their response to pathogens are attributed to the host cell type [20]. Heterogeneity expressed by different pathogens results in various host cell responses, resulting in local or systemic infection. The biological implications of these results are worth exploration with spatial and trajectory information of great value for analyzing the dynamics of host-pathogen interactions. To obtain single cell transcriptomes, traditional isolation of single cells usually digests whole tissues or captures the entire cell [87], failing to preserve spatial context. Recently, pioneering work combining single molecule fluorescence *in situ* hybridization (smFISH) with RNA quantification was used to interrogate spatial gene expression at the single cell level [88]. Subsequently, efforts have been made to increase the number of transcripts measured within a single cell by use of MERFISH [89] and seqFISH+ [90]. Integrating microarray-based spatial transcriptomics can assist in mapping the architecture of scRNA-seq-defined subpopulations [91]. Related computational analysis strategies such as trendsceek [92], which measures single-cell spatial gene expression have been developed as well.

Both infection and immune response are continuous processes while most scRNA-seq samples are taken at discrete time periods. One mean by which to supervise this dynamic process is to implement real-time monitoring, which is technically challenging and expensive. Another solution is the use of computer models to simulate potential cell developmental paths based on similarities in expression patterns, which are referred to as trajectory inference or pseudo-time analysis [93]. Recently, utilizing kinetic scRNA-seq, Abbas *et al.* characterized pDCs activation trajectories during mouse cytomegalovirus (MCMV) infection by pseudo-time analysis [94]. They found pDCs to manifest multiple functions (IFN-I production and T cell activation) in a time and space tightly orchestrated manner. Likewise, Khatun *et al.* evaluated CD4^+^ T cell clonal expansion and differentiation trajectories in response to acute lymphocytic choriomeningitis virus (LCMV) infection [95]. Although a number of analysis technologies and methodologies are available, method consideration is mainly dependent on dataset dimension and trajectory topology [96]. These innovative tools will enable the recall analysis of *in vivo* infections, which will provide for detailed identification of pathogenic mechanism, even though limitations still exist.

## PERSPECTIVES

**Figure 2:**
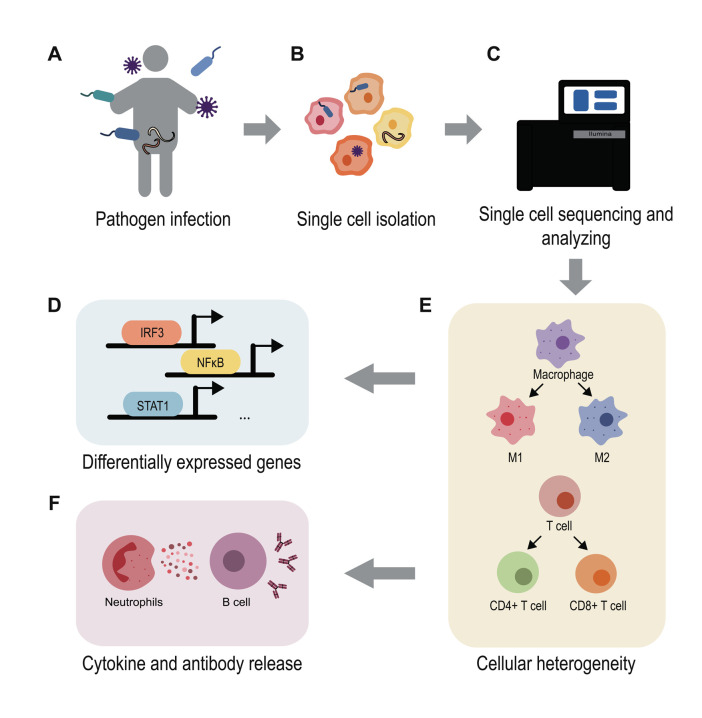
FIGURE 2: Using single-cell RNA-seq to understand host-pathogen interactions. Pathogens like bacteria, viruses, fungi and parasites can infect hosts and cause various infectious diseases **(A)**. Using scRNA-seq methods enable the study of host and pathogen interactions at a high-resolution level **(B-C)**. The single cell transcriptomes reveal the differentially gene expression map during infection **(D)**, demonstrate the cell heterogeneity and immune cell development **(E)**, uncover the mechanism behind the inflammatory and anti-microbial response **(F)**. The application of scRNA-seq highly contributes to infection research.

Advances in the application of scRNA-seq for the study of infections have dramatically broadened our understanding of the molecular details of host-pathogen interactions (**Figure 2**). These advances have allowed for the prediction of infection outcomes and the discovery of new diagnostic biomarkers and therapeutic targets [97]. For example, Ben-Moshe *et al.* developed a deconvolution algorithm for predicting bacterial infection outcomes based on the scRNA-seq data of human PBMCs infected with *Salmonella* [98]. Applying this algorithm to bulk RNA-seq data from cohorts of TB patients during different stages of disease, they not only distinguished active TB patients from healthy individuals but also identified individuals with higher risk for development of active TB. Considering the urgent need for new drugs and vaccines for COVID-19, Alakwaa *et al.* developed a bioinformatic pipeline to prioritize drug candidates based on existing scRNA-seq data and drug perturbation databases, which identified four drugs including didanosine with potential efficacy [99]. Such studies offer advanced treatment options for infectious disease therapy and intervention.

In addition to the spatial transcriptome, which reveals spatial cellular information, emerging multiple-omics single cell analysis can link chromatin and protein features to gene expression in a single cell [100], e.g., scDam and T-seq [101] and SHARE-seq [102]. Combinatorial approaches will further shed light on the multidimensional features of a single cell, refining characterization of the cell heterogeneity during infection. Overall, advances in scRNA-seq technology have greatly improved our understanding of host-pathogen interaction and made fundamental contributions to the development of new strategies for the control of infectious disease [103]. These advances have enabled deeper understanding of the infectious progress, although analyses of these types have just been initiated. Further efforts are still required and with the continuous creation of new approaches, the mechanism of the infectious diseases will gradually be uncovered, as well as new drugs and effective treatments.

## Abbreviations:

COVID-19: – Coronavirus disease 2019;
HC: – healthy control;
IFN: – interferon;
LTBI: – latent TB infection;
Mtb: – Mycobacterium tuberculosis;
NK: – natural killer;
PBMC: – peripheral blood mononuclear cells;
pDC: – plasmacytoid dendritic cell;
scRNA-seq: – single-cell RNA sequencing;
TB: – tuberculosis;
Tfh: – follicular helper T cell;
UMI: – unique molecular identifier.
